# Saliva and Blood Cortisol Measurement in Bottlenose Dolphins (*Tursiops truncatus*): Methodology, Application, and Limitations

**DOI:** 10.3390/ani12010022

**Published:** 2021-12-23

**Authors:** Daniela Rickert, Ralph Simon, Lorenzo von Fersen, Katrin Baumgartner, Thomas Bertsch, Clemens Kirschbaum, Michael Erhard

**Affiliations:** 1 Nuremberg Zoo, 90480 Nuremberg, Germany; daniela.rickert@amberg-mail.de (D.R.); lorenzo@vonfersen.org (L.v.F.); Katrin.Baumgartner@stadt.nuernberg.de (K.B.); 2 Department of Veterinary Sciences, Faculty of Veterinary Medicine, Institute of Animal Welfare, Ethology and Animal Hygiene, Ludwig-Maximilians-University, 80637 Munich, Germany; M.Erhard@tierhyg.vetmed.uni-muenchen.de; 3 Institute of Clinical Chemistry, Laboratory Medicine and Transfusion Medicine, Nuremberg General Hospital, Paracelsus Medical University, 90419 Nuremberg, Germany; thomas.bertsch@klinikum-nuernberg.de; 4 Department of Psychology, Faculty of Science, Institute of General Psychology, Biopsychology and Methods of Psychology, Technische Universität Dresden, 01069 Dresden, Germany; clemens.kirschbaum@tu-dresden.de

**Keywords:** animal welfare, bottlenose dolphin, *Tursiops truncatus*, stress measurement, saliva cortisol, blood cortisol

## Abstract

**Simple Summary:**

Animal welfare assessments in zoological facilities are becoming increasingly important. Two main assessment tools are behavioral observations and stress hormone measurements. At our facility (Nuremberg Zoo), cortisol levels are routinely determined every time blood samples are taken. We can show that the blood cortisol content of bottlenose dolphins depends on the way in which sampling is performed. Cortisol levels are significantly lower when blood samples are taken during voluntary medical training compared to when dolphins are sampled on a lifting platform, which results in higher cortisol levels. For a subset of the blood cortisol data, we simultaneously sampled saliva cortisol. However, we did not find any correlation between saliva cortisol and blood cortisol values. We also tested whether saliva samples are contaminated by fodder fish or diluted by pool water, finding that some fish and squid species exhibit high cortisol values. Consequently, dolphin saliva is highly contaminated directly after feeding, and increased values can be measured up to 4 min after feeding. We recommend being very careful when sampling saliva, and interpreting saliva cortisol values with caution.

**Abstract:**

A central task of zoos and aquaria is the frequent and accurate assessment of their animals’ welfare. Recently, important steps have been made, such as the introduction of animal welfare evaluation tools and welfare decision trees. To determine animal welfare, it is not only important to collect life history data, such as longevity and reproductive success, but also for experienced observers or caretakers to conduct behavioral observations on a regular basis to assess animals’ emotional state. To physiologically validate welfare observations, glucocorticoid levels are usually assessed, as they are a common indicator of stress. While, for many animals, these levels can be easily determined via fecal or hair samples, for cetaceans, the levels are usually determined via blood samples. As blood samples cannot be taken very frequently and the process may cause stress to the animals (if the samples are not taken following medical training), other techniques, such as the measurement of health biomarkers (especially cortisol, which can be measured in saliva), have become the focus of cetacean stress research. However, there are two problems associated with saliva measurements in cetaceans: saliva might either be diluted with pool water or be contaminated by fodder fish, as frozen fish usually contains high levels of cortisol. In our study, we investigated how saliva cortisol levels are connected to blood cortisol levels and how saliva cortisol can be influenced by fodder fish. We examined saliva and blood samples in eleven bottlenose dolphins (*Tursiops truncatus*) kept in an outdoor and indoor facility in Germany. Furthermore, we assessed the cortisol levels of different kinds of fodder fish. Our data show that, although saliva cortisol values are elevated under stress and arousal, they seem not to be correlated with blood cortisol values. We also show that, after feeding, saliva cortisol values are increased up to 100-fold. Our results suggest that saliva cortisol measurements in dolphins have to be conducted and considered with care, as they can easily be contaminated. Moreover, it is important to use the right laboratory method in order to specifically detect cortisol; in our study, we conducted reliable tests, using LC-MS/MS.

## 1. Introduction

Animal welfare—in particular, cetacean welfare—is currently defined along different lines. One approach defines welfare as the balance of the positive and negative affective states [1]. Other approaches focus more on physical conditions, activity patterns, and physiological markers [2,3,4,5]. Currently, different welfare assessment protocols that try to standardize welfare assessments and approach animal welfare more holistically are used.

Since the 1990s, the ‘five freedoms’ have been used as a basis for evaluating animal welfare in livestock farms [6]. Mellor (2016) further developed this idea and pointed out that it is important to minimize the negative experiences of animals and, at the same time, provide opportunities for them to have positive experiences [7]. In order to verify this notion and obtain a more holistic view of the welfare of an animal, specific animal-related criteria must be developed. The so-called C-Well^®^ protocol [8] is one of the first welfare assessment tools developed for bottlenose dolphins (*Tursiops truncatus*); however, it does not include behavioral observations made using a systematic approach. Another protocol is the American Humane [9,10], which is a conservation certification program that consists of the evaluation of the facility and management of animals by experts in the field. The European Association for Aquatic Mammals (EAAM) recently launched the Dolphin WET (Welfare Evaluation Tool) project. Another tool, the welfare decision tree [2], was introduced during the 1st Welfare Workshop from 3 to 5 May 2016 at Nuremberg Zoo. The assessment of the glucocorticoid levels of animals is also part of this decision tree.

Modern stress theory defines stress as a state in which homeostasis is lost [11]. Different stressors are known; they are usually divided into two different groups: physical, such as heat and cold, and psychological, such as fear and frustration. The physical response to various stressors begins in the central nervous system (CNS). In the hypothalamus, incoming nerve impulses are converted into hormonal signals. This is referred to as neuroendocrine coupling [12,13].

The endocrine stress response begins in the paraventricular nucleus of the hypothalamus and then runs via the pituitary gland to the adrenal cortex. Initially, the neurosecretory cells of the nucleus are activated and CRH (corticotropin-releasing hormone) is increasingly produced and secreted. CRH causes the release of ACTH (adrenocorticotropic hormone) in the anterior pituitary. ACTH, in turn, causes the secretion of glucocorticoids from the adrenal cortex. Therefore, the hormonal stress response is also called the hypothalamic–pituitary–adrenal (HPA) axis [14]. Various studies have shown that cortisol secretion in both animals and humans is subject to daily and seasonal fluctuations [15,16,17,18,19,20,21]. In mammals active during the day, the cortisol level rises throughout the morning and falls over the course of the day [22].

Glucocorticoids can be detected in blood; in different body fluids, such as saliva; in the skin; and even in the excreta [14,23] or blow [9]. As in some cases the sampling procedure itself can be a source of stress [24], it would be useful to be able to measure cortisol using less invasive matrices. The use of non-invasive techniques, such as saliva sampling, has become increasingly established, especially in wild animals and zoo animals [25]. Non-invasive techniques are also more frequently used in pets, as blood sampling itself can also lead to an increase in glucocorticoid levels [26]. In some cases, to circumvent stress, animals can be trained to voluntarily allow blood sampling, which then does not require any restriction. Dolphins were among the first zoo animals in which this training was started and perfected. Today, blood sampling is an everyday part of the husbandry routines of these animals.

The primary glucocorticoid that has been identified and studied in dolphins is cortisol [27,28]. First studies on cortisol levels in bottlenose dolphins (*Tursiops truncatus*) mainly used blood tests [29,30,31,32] to measure cortisol. However, it was shown that too-frequent blood sampling in bottlenose dolphins (*Tursiops truncatus*) may not be feasible because of the risk of inflammation.

In other wild animals, feces and urine tests have proved particularly effective [33,34,35,36,37,38]. These investigations are not limited to mammals; reptiles also show stress reactions in the form of increased glucocorticoid levels in the feces [39]. Hair has also been found to be a suitable matrix in humans [40] and other mammals [41,42,43], and feathers have been used in birds [44] in cases where glucocorticoid levels over longer periods were of interest, as cortisol is incorporated throughout the growth phase of hair and feathers [45].

Fecal samples can also be obtained from bottlenose dolphins (*Tursiops truncatus*). This is usually achieved by the catheterization of the rectum [46,47,48,49]. In order to obtain fecal samples, animals must be trained to lie on their backs and remain there. Since the ampoule is not regularly filled with feces, this method is not only more time-consuming but requires additional training and is also difficult to conduct on a regular basis. Urine samples can also be collected through training [50].

A feasible method for frequent, non-invasive sampling in mammals is saliva collection.

The major salivary glands in almost all mammals are the parotid, sublingual, and submandibular glands. These glands, and hundreds of small salivary glands (labial, buccal, palatal, and lingual) secrete saliva with different compositions [51,52,53].

Saliva sampling has been used in different species as an established method for performing fast, frequent, and non-invasive cortisol measurements. The fact that the measured values correlate with the blood cortisol level has been proven for several different species such as goats, sheep, pigs, horses, and dogs [54,55]. Even in free-ranging animals, it is possible to collect saliva, as was shown for rhesus monkeys (*Macaca mulatta*) [25], for example.

Although it is relatively easy to obtain uncontaminated saliva from terrestrial mammals, for aquatic mammals this is much more challenging, because saliva samples can be diluted with pool water. Sampling must, therefore, take place under conditions that exclude or prevent contamination with pool water as far as possible. For this purpose, sites in the mouth that are usually not contaminated with water but still contain enough saliva to allow measurement should be aspirated. Whether pool water is present in the saliva samples obtained is almost impossible to determine. In saliva there are no enzymes, hormones, or salt concentrations, etc., which would always occur in the same form, as it is the case for blood.

Only a few studies have been carried out on the salivary or blood cortisol content of dolphins. These have been conducted with different objectives: A first study with dolphins showed that simultaneous measurements of saliva and blood cortisol showed that saliva cortisol concentrations represent 10% to 27% of the cortisol level in blood [56,57]. Ugaz et al. (2013) published a study on 23 bottlenose dolphins (*Tursiops truncatus*) kept in four different holding facilities. Saliva samples were collected once daily, in the morning, from fasting animals. The dolphins showed salivary cortisol levels between 0.0116 and 1.5109 nmol/L [58]. Furthermore, Monreal-Pawlowsky et al. used the salivary cortisol concentration as a possible welfare indicator [59].

In addition to the dilution problem mentioned above, there is the possibility of the contamination of samples with fodder fish. Dolphins in human care rarely use their teeth to loot-catch. Fish are usually given by keepers into the dolphin’s opened mouth. Dolphins do not chew their food but swallow it whole. When swallowing, the tongue is pressed against the palate. Liquid from fodder fish can enter the mouth of the dolphin and contaminate the saliva sample. This could contaminate the saliva with cortisol from the fodder fish [60,61,62].

In summary, in contrast to terrestrial mammals, saliva sampling in aquatic mammals features particular challenges. Additionally, in our opinion, the correlation between blood cortisol content and salivary cortisol content is not clear.

The primary purpose of this study was to test whether salivary cortisol can be used as a measure to estimate HPA axis activity in dolphins. In the first phase, we tested whether salivary cortisol measurements correlate with time-matched blood cortisol levels.

For this study, we investigated the informative value of saliva cortisol samples under different dilution and contamination scenarios for bottlenose dolphins (*Tursiops truncatus*), and tested how saliva cortisol levels are correlated with blood cortisol levels. We investigated the following hypotheses: (1) saliva can be contaminated by fodder fish that contain high amounts of cortisol, (2) saliva can be diluted with pool water. To exclude contamination with cortisol from fish, we assessed the cortisol contamination of different types of fodder fish and conducted a feeding experiment with five bottlenose dolphins (*Tursiops truncatus*) to demonstrate how a fish meal can influence saliva cortisol values. Moreover, we tested if and how both levels are correlated using simultaneous blood and saliva cortisol measurements.

## 2. Materials and Methods

Animals: During the time in which our study was carried out, 11 bottlenose dolphins (*Tursiops truncatus*) were living at Nuremberg Zoo and data were collected from all of these animals. Table 1 provides an overview of the animals and their individual contributions to the datasets and experiments. The blood samples were all taken due to medical indication in the years 2010 to 2020. The simultaneous blood and saliva sampling was conducted in the years 2018 and 2019. Again, the blood samples were taken because of a medical reason. Almost all blood samples were taken early in the morning. The feeding experiments took place in July and August 2019 (experiment I) and February 2020 (experiment II) and were always started in the morning at 8 am, before the first meal. As we had a very tight sampling procedure, the feeding experiment could only be conducted with certain, well-cooperating individual dolphins.

Housing: The dolphins were housed in an indoor and outdoor facility with three different indoor pools and five outdoor pools, which were connected to each other and had a total volume of salt water of more than 7 million liters. The depths varied from 7 m to 0.5 m. The indoor part featured a lifting platform in a so-called round-pool, which had a double floor. The upper, lattice-shaped floor could be raised automatically so that the animals could be lifted completely or partially out of the water.

The pools contained a closed life support system with ozone and a small amount of chlorine as a disinfectant. The animals were fed with different types of fish and cephalopods (herring (*Clupea*), mackerel (*Scomber scombrus*), capelin (*Mallotus villosus*), sardines (*Sardina pilchardus*)*,* sprat (*Sprattus*), and squid (*Loligo* spp.)). Training was based on positive reinforcement. All the animals were trained for different behaviors to facilitate/allow medical inspection, diagnostics, or treatment. A staff of eleven full-time employed qualified zookeepers took care of the animals.

**Figure 1:**
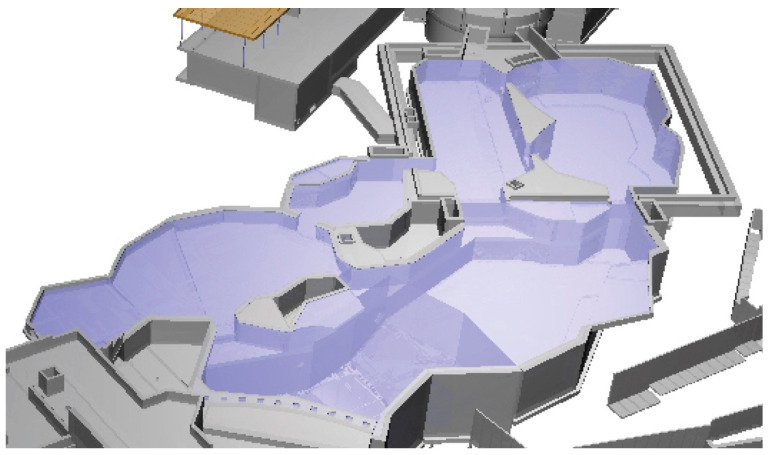
Scheme of the outdoor pools, called the lagoon, where the dolphins were housed.

The water temperature was usually between 17 °C and 20 °C, during summer it sometimes went higher but never above 25 °C. In winter the water temperature was always kept above 14 °C. Down to an outside air temperature of −4 °C, the animals had the possibility to freely choose between inside and outside pools. As soon as the air temperature permanently fell below −5 °C, the animals were kept indoors. To enlarge the heated indoor space during winter an air dome was placed over both upper pools (see Figure 1). The air temperature at the indoor facilities was kept higher than the water temperature and was at least 15 °C.

Saliva sampling method: We established our saliva sampling protocol based on the following considerations. All sampling procedures were carried out while the animal remained in the pool. In this way, it could be ensured that repeated sampling procedures could be conducted with every animal at any place in the pool. At Nuremberg Zoo, the dolphins are trained to allow several medical behaviors, including saliva sampling. To take a saliva sample, the animals had to stretch their heads out of the water, open their beaks, and keep them open while the sample was taken until the reward whistle (secondary reinforcer) was blown.

Our second consideration was to find the best place to take saliva samples in the mouth of the dolphin. Salivary glands end in the whole mouth, but according to our experience watery accumulations, which could be saliva or pool water, often occur at the base of the tongue. Sampling on the palate was not possible because there was not enough saliva available. It turned out that the fluid obtained at the side of the tongue was more viscous than the fluids obtained elsewhere. Therefore, we assumed that the saliva in this region is the least diluted. For our method, it was important that the fluid obtained remained comparable and, therefore, we chose the side of the tongue as the best sampling area.

**Figure A1:**
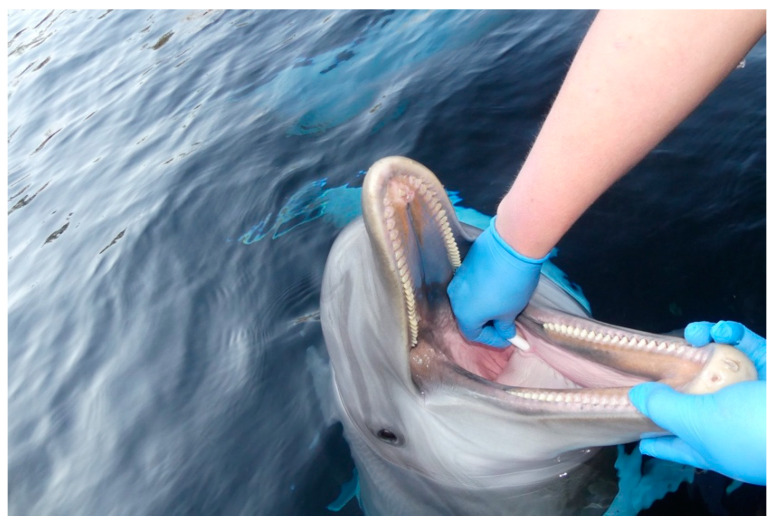
Saliva sampling, Image of the saliva sampling procedure.

The specially designed Sarstedt “Cortisol-Salivette” (order number: 51.1534.500) was used for saliva sampling. This consists of a 4.2 cm-long roll of synthetic wadding that is inserted into a plastic container containing a small hole at the bottom and is located in a plastic tube. After taking the sample, the centrifugation of the Salivette device results in low-viscosity saliva at the bottom of the tube. The zookeepers who took the samples followed the instructions listed in the Appendix A. For sampling, the swab was moved back and forth on the side of the tongue for 5 s (see Figure A1). After sampling, the samples were centrifuged for 5 min at 5000 rpm in the veterinary laboratory, and then the saliva remained in the tube was stored at −20 °C or lower.

Laboratory methods used to analyze saliva: the swabs were tested for their cortisol content using the following methods: Liquid chromatography coupled with tandem mass spectrometry (LC-MS/MS): Cortisol levels were quantified by liquid chromatography coupled with tandem mass spectrometry, as described in detail elsewhere [63]. The intra- and interassay coefficients of variation are below 10% for this method. These samples were examined in the laboratory of the Chair of Biopsychology, Technical University Dresden.

**Figure 2:**
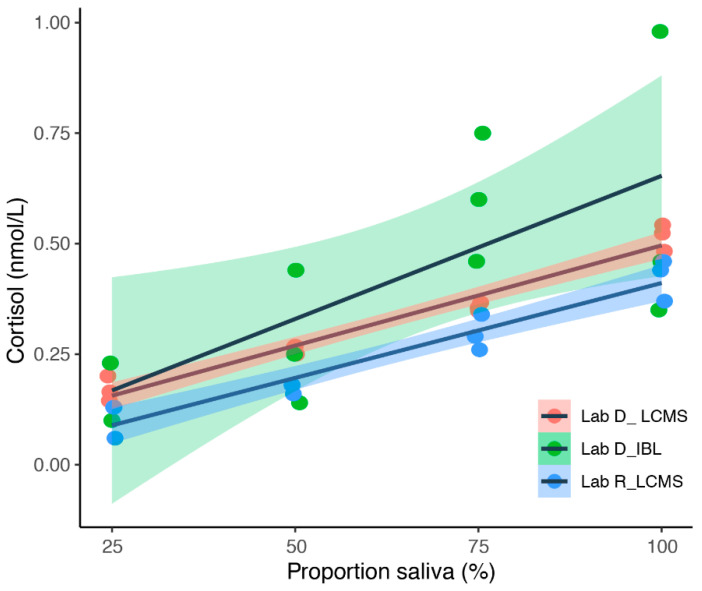
Dilution series for saliva cortisol analyzed at two laboratories (Lab R and Lab D). Saliva was collected over some days, accumulated, and then diluted with different proportions of 0.9% NaCl solution. Saliva samples were analyzed with two different methods LC-MS/MS (LC-MS) and immunoassays (IBL).

Validation of the saliva cortisol measurement method: Since there are no validated tests for measuring salivary cortisol concentrations in dolphins, we had to find a way to validate the laboratory methods ourselves. For this, we used a series of diluted saliva samples from one bottlenose dolphin (*Tursiops truncatus*, Moby). In 2017, samples were collected, frozen, and pooled over some days in order to obtain a good quantity of saliva. The saliva was then defrosted, divided, and diluted with 0.9% NaCl solution to create a dilution series as follows: 100% saliva, 75% saliva/25% NaCl, 50% saliva/50% NaCl, 25% saliva/75% NaCl, 100% NaCl. The diluted samples were again divided into 60 cuvettes (12 cuvettes for each concentration level) and sent blinded to 4 different laboratories (Lab names were anonymized; we will use the following names: Lab B, Lab E, Lab D and Lab R). Each laboratory received three cuvettes of each concentration level. With this dilution series, we aimed to find out which laboratory methods were most suitable for measuring the correct cortisol content on a relative scale. The laboratories worked with different methods: Two laboratories worked with LC-MS/MS (Lab D and Lab R). Lab D additionally worked with a CLIA (Chemiluminescence Immunoassays (CLIA) from IBL international, now Tecan), a specially designed test for the detection of cortisol in human saliva. Lab E used the same CLIA kit from IBL and Lab B worked with an EIA (Enzyme Immunoassay (EIA) Cortisol EIA Detection kit, Neogen Europe, Ayr, UK), a test for the quantitative analysis of cortisol levels in biological fluid. We expected that all laboratories would give comparable results for samples with identical concentrations and that the readings would reflect the dilution series. We also expected that cortisol would not be measurable in the samples that did not contain saliva. This expectation was met only by laboratories working with the LC-MS/MS method (Lab D and Lab R). Lab B and Lab E were not able to detect anything and, therefore, could not provide any results. Lab D was able to display the dilution series correctly, both with the CLIA and the LC-MS/MS assay; however, for the CLIA the values varied greatly within the same dilution level (see Figure 2). Lab R, which worked with LC-MS/MS provided similar results as Lab D. Fitting a linear regression model showed that only 46.5% of the variation in cortisol levels could be explained by the proportion of saliva involved (LM, F_1,10_ = 7.815; *p* = 0.0209); see Figure 2. Fitting a linear regression model to the LC-MS/MS, however, showed that 92.3% or 96.1% of the variation in cortisol levels could be explained by the proportion of saliva involved (Lab R: LM, F_1,10_ = 120.1; *p* < 0.001; Lab D: LM, F_1,10_ = 243.8; *p* < 0.001; see Figure 2). We analyzed all subsequent samples with the LC-MS/MS method at the Lab D, the laboratory of CK in Dresden, Germany.

Blood collection procedure: Blood samples are taken regularly as part of routine medical practice. The frequency at which they are taken depends on the age and health status of the animals. When taking blood via training, the dolphin presents its fluke out of the water on demand. The animal remains under signal control until the blood collection procedure is finished. However, the animal can leave the situation at any time by swimming away. As mentioned before, the dolphins are trained to allow several medical behaviors. The animals are trained to be handled on a lifting platform on almost a daily basis. In cases where blood sampling must be carried out under restraint conditions, the dolphin will lie on a soft mattress and be restrained by their trainers, who position themselves on both sides of the dolphin.

Laboratory methods for evaluating blood: The blood samples were all examined in the laboratory of the Institute of Clinical Chemistry, Laboratory Medicine and Transfusion Medicine—Nuremberg General Hospital. The cortisol value was also routinely determined, even if the reason for the examination was always different. Cortisol in heparinized plasma or serum was measured until the end of October 2015 using the ‘Elecsys Cortisol’ assay from Roche Diagnostics (Roche Diagnostics, Mannheim, Germany). From November 2015, the ‘Elecsys Cortisol II’ assay from Roche Diagnostics was used for analysis. According to the manufacturer, the ‘Elecsys Cortisol II’ assay showed approx. 20% lower cortisol concentrations. Until September 2011, only whole numbers were used for statistics, as only whole numbers were displayed as results in the laboratory information system (LIS). Afterwards numbers with one decimal place were displayed in the LIS and used for statistics. The lowest value transferred from the laboratory instrument to the LIS was 0.1 µg/dL.

**Figure 3:**
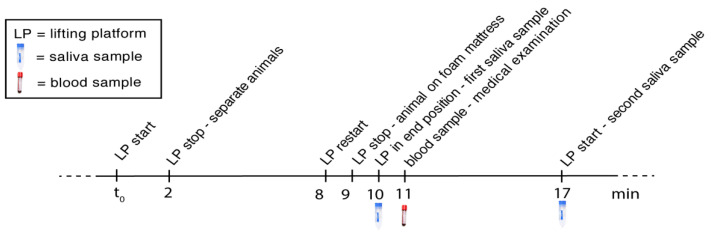
Exemplary sampling scheme for the simultaneous blood and saliva sampling.

Comparison of cortisol in saliva and blood: In order to compare the correlation between blood and saliva cortisol, both parameters were assessed for four animals during different medical examinations (for example, X-rays). A single blood sampling procedure and multiple saliva sampling procedures were always used. The time at which the blood and saliva samples were taken was recorded precisely; see Figure 3.

Cortisol contamination by fodder fish: As previous studies have shown, the fishing method (rod or line) used can lead to a significant increase in cortisol in many fish species, for example, red snappers (*Pagrus auratus*) [64]. Therefore, we assumed that the fodder fish given to dolphins may also contain significant levels of cortisol. In order to verify this assumption, the cortisol content of the thawing water of various feed fish species and cephalopods was examined by LC-MS/MS (herring (*Clupea*), mackerel (*Scomber scombrus*), capelin (*Mallotus villosus*), sardines (*Sardina pilchardus*), and squid (*Loligo.* spp.)). We thawed a complete palette of fish and squid, collected the thawing water, and stirred it. This prevented the formation of phases (fish mucus and water). We took samples by immersing a Salivette in the thawing water and then treated it as we would a saliva sample. We used different pack sizes for different fish species. We took one sample each from 20 kg of Atlantic herring, 15 kg of Baltic herring, 20 kg of mackerel, 5 kg of capelin, 8 kg of squid, and 8 kg of sprat.

Feeding experiment I: To measure the impact of fodder fish on saliva cortisol values, we took three samples before reinforcement and one sample directly after the dolphins swallowed the fish. Saliva samples were taken from three dolphins (Sunny, Donna, and Dolly) each morning between 8:00 and 8:15 a.m. according to the described sampling protocol. In total, 128 samples were taken. Only six samples could not be used for analysis, as they were either empty or had insufficient amounts of saliva. In order to exclude a possible dilution effect by water, the experimental setup was defined as follows: The animals were not fed before sampling. They were called individually to the border of the pool, after which they opened their mouth on command. The idea was to offer ice cubes or gelatin cubes to the animals so that the remaining water would be swallowed by ingesting the cubes. Only if the mouth was apparently free of water a saliva swab was taken. Then, the animal was sent away. About one minute later, it was called again and the second swab was taken under the described conditions. We continued to take a third swab in the same way. Only after the third sampling the fish was given as a reward. A fourth sample was taken after the reward to detect the possible influence of the fodder fish. To take the samples, the animals were divided into several groups at short notice so that one trainer could always take care of one animal. Otherwise, the daily routine was not modified.

**Figure 4:**
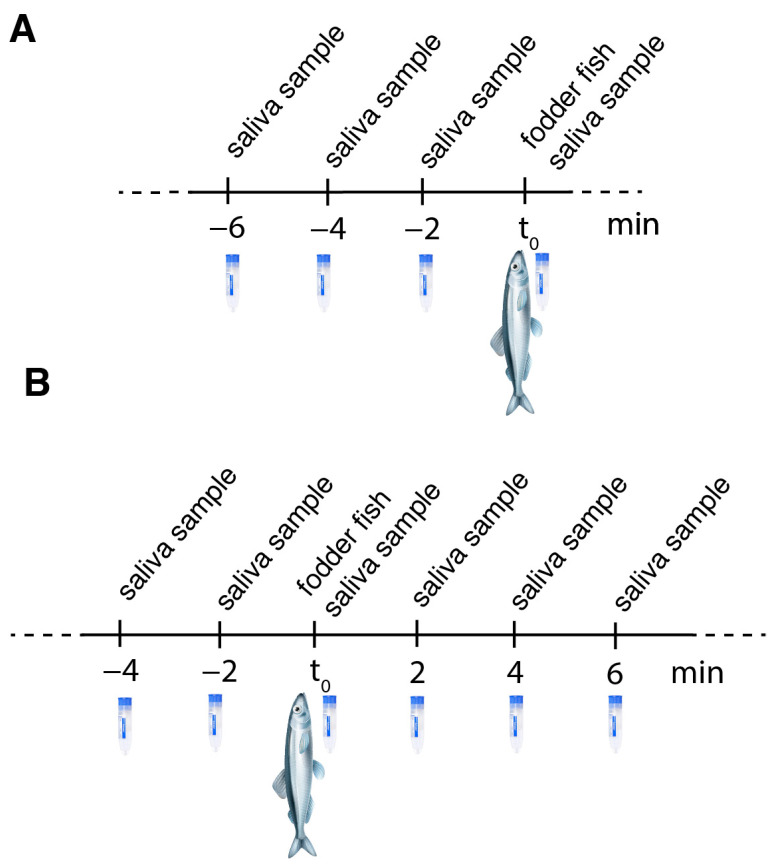
Fixed saliva sampling schemes used for (**A**) feeding experiment I and (**B**) feeding experiment II.

Feeding experiment II: We were further interested in whether the values of cortisol in the thawing water of the fish correlated with the values in dolphin saliva. At the same time, we wanted to find out whether the salivary cortisol value quickly returned to normal after the fish were given. Therefore, the experimental set-up was modified as follows: The first two samples were taken without fish being given as a reward; after the second sample was taken, the dolphin got a reward of five capelin (*Mallotus villosus*) (ca. 150–200 g per fish); directly after this, the third sample was taken. Afterwards, three more samples were taken up to 6 min after feeding, for which the animals were sent away and recalled as before, again without receiving fish as a reward; they received ice cubes or gelatin as a reward instead (see Figure 4). In order to exclude the possible effect of the gelatin obtained from pork rind, the gelatin was also sampled daily. Five female dolphins (Anke, Dolly, Donna, Jenny, and Sunny) took part in this experiment. Two saliva samples were taken before feeding, one with feeding, and three more samples after feeding. We took a total of 180 saliva samples.

Statistics: Statistical analysis was conducted in Rstudio (Version 1.2.5042, Rstudio, Inc.) using the lme4 package by Bates et al. [65]. As the limit of detection for saliva cortisol was 0.02 nmol/L, we substituted measured values below the detection limit with 0.02 nmol/L. For the comparison of the blood cortisol levels between the treatments (training or restriction), we fitted a linear model with treatment, health status, and test assay as fixed effects and animal ID as a random factor (log(cortisol) ~ treatm × health × assay + (1 | dolphin)). For every parameter, we constructed a null model that excluded one fixed factor and included animal ID as a random intercept. We used ANOVAs to compare the null models to the models that included the respective fixed factor. To test the correlation between the blood and saliva cortisol values, we fitted a linear model. We also did this for the dilution series. To test the effect of feeding with fish (Capelin, *Mallotus villosus*) on the saliva cortisol values during the feeding experiments, we used restricted maximum likelihood to fit a linear mixed model with sample time as a fixed factor and animal as a random factor. We also constructed a null model that excluded the fixed factor and used ANOVAs to compare the null models to the models that included the fixed factor. We subsequently conducted Tukey’s post hoc tests for the pairwise comparisons of samples. Data preparation and the calculation of basic statistical parameters (mean, standard deviation, median) were performed in Microsoft Excel. Graphs were generated using MATLAB_R2019a from Mathworks or in Rstudio using the ggplot package from Wickham [66].

## 3. Results

### 3.1. Blood Cortisol Values under Two Different Conditions

We collected blood samples during medical examinations under two different conditions: during a normal medical training procedure and on a lifting platform in a situation where dolphins were restrained. In 251 blood samples taken during training from five females and five males, the blood cortisol values varied between 0.1 µg/dL and 3.9 µg/dL, with an average of 0.75 ± 0.65 µg/dL. If the samples were taken during restriction (n = 208), the cortisol values varied from 0.2 µg/dL up to 9 µg/dL, with an average of 2.71 ± 1.99 µg/dL.

**Figure 5:**
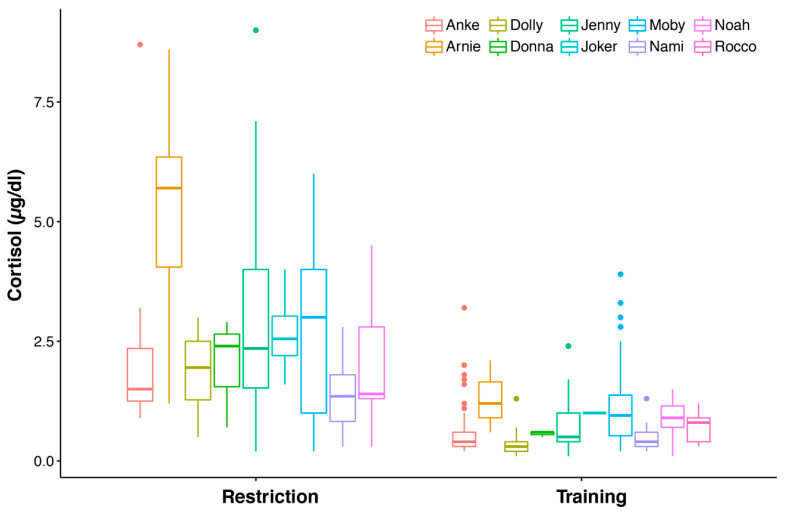
Blood cortisol values for five female and five male dolphins for the treatments restriction, and training.

**Figure A2:**
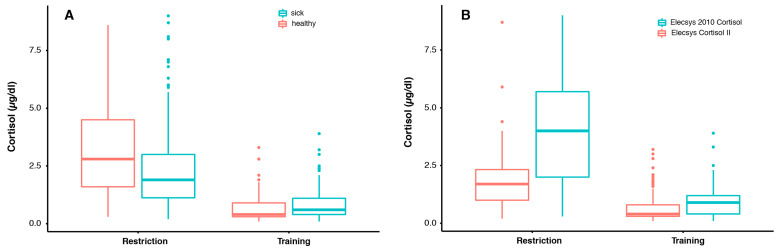
Blood cortisol values for five female and five male dolphins who underwent two different treatments: restriction and training. (**A**) Healthy animals versus animals experiencing medical treatment (referred to as sick). (**B**) The two different test assays, which had different sensitivities.

We used different assays with slightly different standardizations and some blood samples were taken from animals that had health issues and had medication (n = 116). However, we never used any glucocorticoids as a medical treatment. If glucocorticoids are given, one has to be careful, as there is an attenuating effect of dexamethasone administration on cortisol release [67]. We fitted a linear mixed-effects model with treatment, assay, and health status as fixed factors and animal ID as a random factor and compared (ANOVA) it to a null model with only test assay and health status as fixed factors. We found that the treatment (restriction or training) had a significant effect and that all animals’ blood cortisol levels were consistently higher when they were restricted than they were during training (LMM, χ^2^(1) = 169.61, *p* < 0.0001; see Figure 5 and Table A1). Meanwhile, health status was found to have no effect (LMM, χ^2^(1) = 0.4092, *p* = 0.5224; see Figure A2A). The assays also had a significant effect on the blood cortisol values, but this was lower than the training effect (LMM, χ^2^(1) = 32.948, *p* < 0.0001; see Figure A2B).

### 3.2. Simultaneous Blood and Saliva Sampling

To find out how blood cortisol levels are reflected in the salivary cortisol, we tested saliva samples before and after we took a blood sample during times when the animals were restricted.

**Figure 6:**
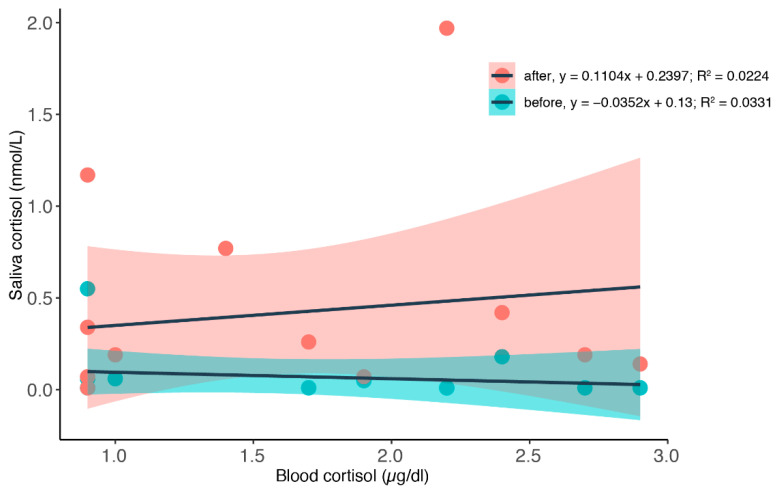
Correlation between blood cortisol values and saliva values during restriction. A linear curve was fitted (see Figure A3 and Figure A4 for other curves). Saliva samples were taken before (green dots) and after (red dots) the blood samples; see Figure 3 for an exemplarily sampling scheme. Three dolphins were sampled (Jenny = 9 samples; Moby = 1 sample; Anke = 1 sample; Nami = 2 samples before, 3 samples after).

We calculated a linear regression to predict saliva cortisol values based on blood cortisol values during restriction; see Figure 6. We did not find significant regression equations for either the saliva values sampled before with an R^2^ of 0.033 or for the values sampled after the blood values with an R^2^ = 0.022 (before: F_1,11_ = 0.3769, *p* = 0.5517; after: F_1,12_ = 0.2754, *p* = 0.6093). We did, however, notice that the saliva samples taken after the blood samples had significantly increased values (before: 0.075 ± 0.150 nmol/L; after: 0.382 ± 0.564 nmol/L. Wilcoxon signed rank test with continuity correction: V = 78. *p*-value = 0.02524). We suspect that the increase in salivary cortisol levels is related to the stress caused by the restraint used during the blood sampling procedure.

### 3.3. Cortisol Contamination of Fodder Fish

**Figure 7:**
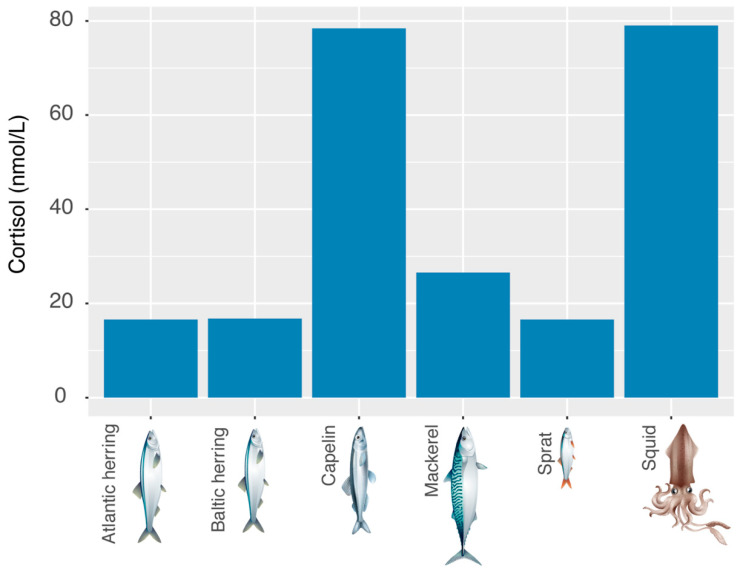
Cortisol values of different fodder fish and cephalopods. We measured samples taken from the thawing water of a 20 kg block of Atlantic herring, a 15 kg block of Baltic herring, a 5 kg block of capelin, a 20 kg block of mackerel, an 8 kg block of sprat and an 8 kg block of squid.

We examined thawing water of packs of frozen fodder fish and cephalopods after a night of them being stored at room temperature. We found the highest mean cortisol concentration values in capelin (*Mallotus villosus*) and squid (*Loligo.* spp.) (see Figure 7).

### 3.4. Feeding Experiment I

**Figure 8:**
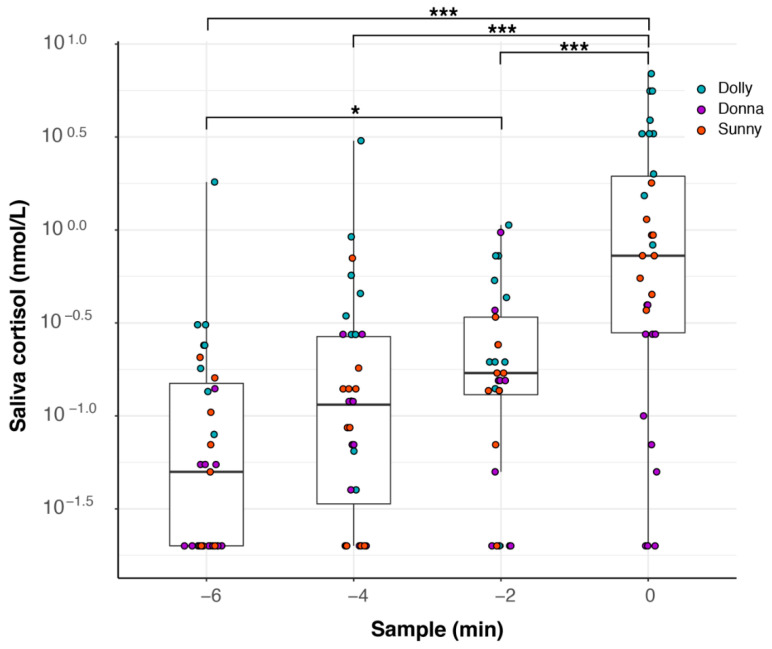
Saliva cortisol values during feeding experiment I. Saliva samples were taken every 2 min over a course of 6 min. At minute 0, dolphins were fed with fish (capelin, *Mallotus villosus*) and a saliva sample was taken directly afterwards. The experiment was conducted with 3 female dolphins and repeated 10–11 times (for Dolly and Donna, 11 times; for Sunny, 10 times). Some selected significance levels of Tukey’s pairwise post hoc comparisons are indicated as follows: * *p* < 0.05, *** *p* < 0.001. For all results, see Table A2.

We found that the fodder fish had a significant effect on the salivary cortisol values (LMM, χ^2^(3) = 54.411, *p* < 0.0001). Indeed, the fodder fish sample differed significantly from all the samples taken before. Interestingly, sample 1 and 3 also differed significantly from each other (pairwise post hoc comparisons between all samples; see Table A2 in the Appendix A for all pairwise comparisons). For the results, see Figure 8.

### 3.5. Feeding Experiment II

**Figure 9:**
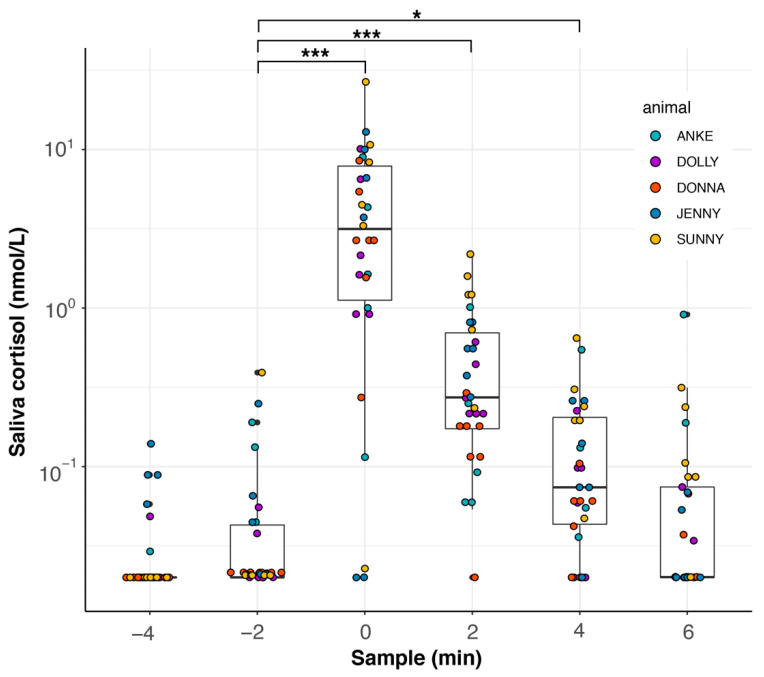
Saliva cortisol values during feeding experiment II. Saliva samples were taken every two minutes over a course of 10 min. At minute 0, dolphins were fed with fish (capelin, *Mallotus villosus*) and a saliva sample was taken directly afterwards. The experiment was conducted with 5 female dolphins and repeated 5–7 times (Anke, 5 times; Dolly, Jenny, and Sunny, 6 times; Donna, 7 times). Some selected significance levels of Tukey’s pairwise post hoc comparisons are indicated as follows: * *p* < 0.05, *** *p* < 0.001. For all results, see Table A3.

To measure how increased saliva values from fodder fish are diluted after feeding, we carried out a second feeding experiment where we also measured saliva values up to 6 min after feeding. We found a significant effect of feeding on salivary cortisol values (LMM, χ^2^(5) = 189.63, *p* < 0.0001), which was mainly caused by the fodder fish given at sample minute 0; see Figure 9. The salivary cortisol levels peaked at the time of feeding and decreased thereafter, returning to pre-feeding levels 6 min after feeding. Using pairwise post hoc comparisons between sample −2 and all following samples, we found significant differences for sample 0 (*p* < 0.0001), sample 2 (*p* < 0.0001), and sample 4 (*p* = 0.0016), but not for sample 6 (*p* = 0.9412); see Table A3 for all pairwise comparisons.

## 4. Discussion

The analysis of blood samples for the quantification of various hormones is an established method, providing fast and accurate results. Disadvantages include the fact that blood sampling is always an invasive procedure and that the values measured can be influenced by the blood sampling method used (via training or restriction). Blood sample results are available quickly and their analysis is comparably inexpensive. Blood is a good matrix by which to obtain valid results quickly for routine sampling. However, it becomes difficult if the samples have to be taken more frequently (e.g., several times a week or several times a day at different times). Various factors can have an influence on the amount of cortisol in the blood (e.g., social stress [68], cold stress, daytime, and season [69]). It can clearly be shown that blood collection on the lifting platform leads to significantly higher values; hence, it can be assumed that even if the situation is familiar to the animals, it is likely to lead to higher stress levels. The predictability of the situation plays a role here. The dolphins know their routines exactly. In training, the animals can choose whether they want to participate or not. At any time, they are the masters of the situation, which they can end by simply swimming away.

The situation on the lifting platform (which is also familiar), however, is completely different. Here, the animals are restrained because they cannot swim away as soon as the lifting platform is above a certain water level. This leads to them experiencing significantly more stress, which leads to higher cortisol levels. Our measured blood values may show that the predictability of a situation and the possibility of avoiding it by one’s own actions significantly influence the blood cortisol level. The different blood values obtained are mainly dependent on the type of blood sampling used (restriction/training) and also differ slightly between individuals; however, we found no dependency on the physiological condition of the animal (sick/healthy).

Salivary cortisol levels are used more and more to measure stress levels in zoo animals. While this appears to be a relatively straightforward approach for studying species such as non-human primates [70,71], dogs, cats, and others [72,73,74], the investigation of aquatic animals using saliva as a non-invasive biomarker matrix is more challenging. Different factors can lead to invalid results here, since saliva samples may be contaminated/diluted by pool water and/or fodder fish. The aim of our study was to investigate whether these factors are common downsides of salivary cortisol measurements. We demonstrated that the contamination of saliva with fodder fish had a significant effect on the cortisol levels measured. This influence persisted after the fish had been swallowed; in our measurements, this was still measurable up to about 4 min after the feed was given. Therefore, salivary samples should never be taken while animals are fed or shortly after a feeding reward.

Why are the saliva cortisol levels not correlated to the blood cortisol levels? One reason for the missing correlation could be that saliva is formed and secreted in different glands, this means that the saliva composition is variable. After a study by Vincent and Michell, it was shown that in dogs exposed to acute stress, the salivary cortisol value increases almost parallel to the blood cortisol value. An increase was observed 10 min after the onset of stress [54]. So why couldn’t we find parallel increase in dolphins? One reason for the different cortisol values found in blood and saliva could be that there is always the possibility of dilution by pool water even when taking samples on a lifting platform or the comparatively short time interval between blood and saliva sampling. Additionally, the measurement of very small amounts of cortisol is associated with some difficulties. This is shown by the fact that two laboratories could not measure any valid values. Another reason could be that the saliva samples were measured by LC-MS/MS, while the blood samples were measured by CLIA: CLIA is an immunological method that is based on concurrent binding of the hormone to the antibody. Because steroid hormones are chemically/structurally similar, antibodies cannot distinguish between steroids with absolute specificity. According to the manufacture instructions, Roche cortisol II for Cobas, the estimated cross-reactivity for corticosterone is 6.58% and the one for 11-Deoxycortisol is 4.9%. In addition to that, antibodies cannot distinguish between free and protein-bound hormone fractions. Cortisol levels in serum measured by CLIA reflect total cortisol, both free and bound. The majority of the blood-cortisol circulates bound to corticosteroid-binding globulin (CBG) and albumin. Normally, less than 5% of circulating cortisol is free (unbound). In saliva, the cortisol is mostly unbound. The LC/MS-MS measures cortisol with an extremely good specificity with almost zero cross-reactivity with other substances. This could also be an explanation for the poor correlation we observed.

Our data show that the exclusive use of cortisol, especially if only quantified on one occasion, is a poor indicator for the general well-being of bottlenose dolphins (*Tursiops truncatus*). It also has to be considered that absolute values of a single sample are not indicators for the condition of an animal. Any sampling must take into account whether the type of sampling used may have an influence on the result. Additionally, the condition of the animal should also be included in the evaluation. This means that the determination of cortisol values alone is not sufficient. In order to be able to make statements about the well-being of animals, one still needs additional information (for example, the health status of an animal) and, perhaps, also behavioral observations, as qualitative and quantitative analyses of behavior were shown to be important welfare indicators in bottlenose dolphins under professional care [75].

We also showed that fish as a reward had an enormous influence on the cortisol content of dolphin saliva. This is because when the dolphins swallow fish, they press it against their hard palate with their tongue and move it towards the gullet, which causes the fish to be “squeezed out”. The body fluids of the fish mix with the saliva and thereby increase the salivary cortisol content. This increase is still measurable 4 min after the reward is given.

Although our data show that the use of saliva cortisol values seem to be rather unreliable because there is no correlation with the blood values, as well as the fact that there can be an influence by dilution with pool water and contamination with fodder fish, we also find indications of significantly increased values due to stress or arousal. In blood versus saliva measurements, we found increased values for the second measurement, where the animals had already been under restriction for some time. In the third sample of feeding experiment I, which was taken before feeding, the cortisol levels were significantly increased. This could possibly point towards a state of arousal or anticipation of food. Therefore, the use of saliva cortisol values might still allow us to obtain information about the animals’ welfare and emotional state. However, samples have to be taken with care, and it has to be considered that their variation is large. Further studies must find suitable sampling schemes for reliable saliva cortisol measurements in aquatic mammals.

## 5. Conclusions

Blood cortisol measurements can be used to measure acute stress. This is clearly demonstrated by the measurements of blood cortisol levels taken on the lifting platform. The non-invasive sampling of saliva is another option for cortisol measurement, but it is more prone to error. This is because, unlike blood samples, saliva samples from aquatic mammals are likely to be contaminated; therefore, care must be taken that the sample is as clean as possible (i.e., without the influence of dilution by surrounding pool water).

Other parameters (especially behavioral observations) also have to be used when assessing animals’ well-being. Basing decisions exclusively on cortisol levels could, as our data show, lead to false results. On the other hand, it is also possible to find important signals in the salivary cortisol levels. Therefore, we think that further research on salivary cortisol in marine mammals is needed.

## Figures and Tables

**Table 1:** Overview of the study animals and the tests and experiments they participated in.

Name	Sex	Born	Birth Year	Blood Cortisol	Blood vs. Saliva Cortisol	Feeding Experiment I	Feeding Experiment II
Anke	female	wild	ca. 1983	×	×	×	-
Arnie	male	captive	2000	×	-	-	-
Jenny	female	wild	ca. 1987	×	×	×	-
Moby	male	wild	ca. 1960	×	×	-	-
Noah	male	captive	2000	×	-	-	-
Rocco	male	captive	2005	×	-	-	-
Nami	female	captive	2014	×	×	-	-
Dolly	female	captive	2007	×	-	×	×
Donna	female	captive	2007	×	-	×	×
Sunny	female	captive	1999	-	-	×	×
Joker	male	captive	1991	×	-	-	-

**Figure A3:**
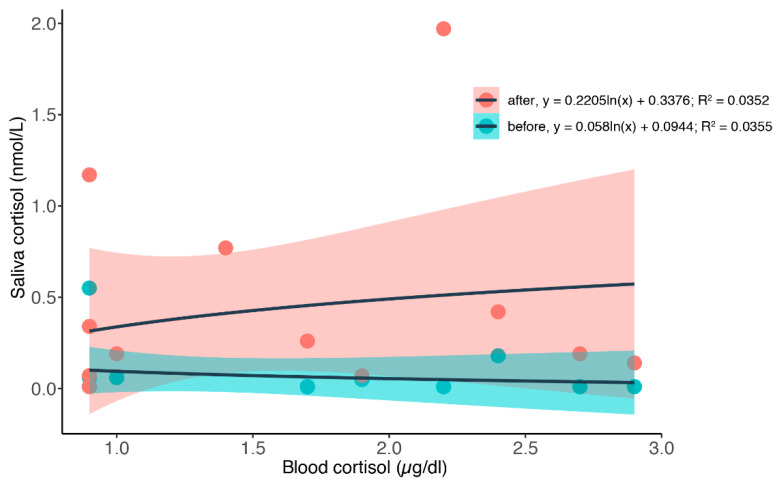
Correlation between blood cortisol values and saliva values during restriction. A logarithmic curve was fitted. Saliva samples were taken before (green dots) and after (red dots) the blood samples; see Figure 3 for an exemplarily sampling scheme. Three dolphins were sampled (Jenny = 9 samples; Moby = 1 sample; Anke = 1 sample; Nami = 2 samples before, 3 samples after).

**Figure A4:**
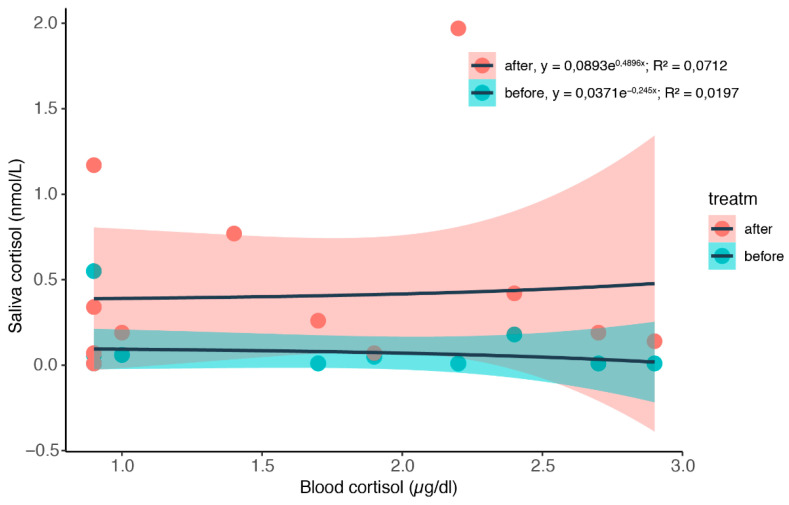
Correlation between blood cortisol values and saliva values during restriction. An exponential curve was fitted. Saliva samples were taken before (green dots) and after (red dots) the blood samples; see Figure 3 for an exemplarily sampling scheme. Three dolphins were sampled (Jenny = 9 samples; Moby = 1 sample; Anke = 1 sample; Nami = 2 samples before, 3 samples after).

## Data Availability

The data presented in this study are available on request from the corresponding authors. The data are not publicly available because they are owned by the owner of the animals and are only available with permission.

## Appendix A

**Instructions for zookeepers:**

Zookeepers who took the samples followed these instructions/this protocol:

- Do not feed the dolphin before sampling.
- Wear gloves.
- Remove the cotton swab from the plastic tube.
- Collect saliva from the side of the tongue for 5 s.
- Put the swab back into the tube.
- Record the date, time of the collection, and name of the animal on the plastic tube.
- If more than one saliva sample is taken, reward the animal with ice cubes or gelatin (only if necessary). If available, take notes on the documentation sheet.
- Send tubes to the veterinarians.

**Table A1:** Statistical results of the linear mixed-effect models (LMMs) fit by REML. The results show the effect of the treatment, test assay, and health status on the blood cortisol levels. Formula: log(Cortisol) ~ treatm + assay + health + (1 | dolphinID). Significance levels are indicated * *p* < 0.05 and *** *p* < 0.001.

LMM-Model	Observation Periods	Estimate	Std. Error	Df	T-Value	*p*-Value
*Blood cortisol*	Intercept	0.38350	0.14475	16.75493	2.649	0.017 *
REML	Treatment training	−1.08618	0.07620	464.74523	−14.253	<2 × 10^−16^ ***
AIC: 1008.779	Elecsys Cortisol III	0.45200	0.07766	461.23621	5.820	1.1 × 10^−8^ ***
	Health status sick	0.04378	0.06810	463.24528	0.643	0.521

**Table A2:** Tukey’s pairwise post hoc comparisons for feeding experiment I; see Figure 8.

Contrast	Estimate	SE	df	T Ratio	*p*-Value
−6 effect vs. −4 effect	−0.595	0.287	122	−2.074	0.1675
−6 effect vs. −2 effect	−0.937	0.294	122	−3.185	0.0098
−6 effect vs. 0 effect	−2.305	0.291	122	−7.911	<0.0001
−4 effect vs. −2 effect	−0.342	0.292	122	−1.173	0.6451
−4 effect vs. 0 effect	−1.71	0.289	122	−5.916	<0.0001
−2 effect vs. 0 effect	−1.368	0.297	122	−4.614	0.0001

**Table A3:** Tukey’s pairwise post hoc comparisons for feeding experiment II; see Figure 9.

Contrast	Estimate	SE	df	T Ratio	*p*-Value
−4 effect vs. −2 effect	−0.242	0.289	179	−0.838	0.9601
−4 effect vs. 0 effect	−4.339	0.289	179	−15.012	<0.0001
−4 effect vs. 2 effect	−2.492	0.289	179	−8.623	<0.0001
−4 effect vs. 4 effect	−1.19	0.289	179	−4.119	0.0008
−4 effect vs. 6 effect	−0.51	0.292	179	−1.749	0.5012
−2 effect vs. 0 effect	−4.097	0.289	179	−14.174	<0.0001
−2 effect vs. 2 effect	−2.25	0.289	179	−7.785	<0.0001
−2 effect vs. 4 effect	−0.948	0.289	179	−3.281	0.0155
−2 effect vs. 6 effect	−0.268	0.292	179	−0.919	0.9412
0 effect vs. 2 effect	1.847	0.289	179	6.389	<0.0001
0 effect vs. 4 effect	3.148	0.289	179	10.894	<0.0001
0 effect vs. 6 effect	3.829	0.292	179	13.131	<0.0001
2 effect vs. 4 effect	1.302	0.289	179	4.504	0.0002
2 effect vs. 6 effect	1.982	0.292	179	6.798	<0.0001
4 effect vs. 6 effect	0.68	0.292	179	2.333	0.1863
